# Galileo: Three-dimensional searching in large combinatorial fragment spaces on the example of pharmacophores

**DOI:** 10.1007/s10822-022-00485-y

**Published:** 2022-11-24

**Authors:** Christian Meyenburg, Uschi Dolfus, Hans Briem, Matthias Rarey

**Affiliations:** 1 Universität Hamburg, ZBH - Center for Bioinformatics, Universität Hamburg, Bundesstraße 43, 20146 Hamburg, Germany; 2 Research & Development, Pharmaceuticals, Computational Molecular Design Berlin, Bayer AG, Building S110, 711, 13342 Berlin, Germany

**Keywords:** Fragment-based drug design, Evolutionary algorithm, Pharmacophore matching, Molecular optimization, Fragment evolution

## Abstract

Fragment spaces are an efficient way to model large chemical spaces using a handful of small fragments and a few connection rules. The development of Enamine’s REAL Space has shown that large spaces of readily available compounds may be created this way. These are several orders of magnitude larger than previous libraries. So far, searching and navigating these spaces is mostly limited to topological approaches. A way to overcome this limitation is optimization via metaheuristics which can be combined with arbitrary scoring functions. Here we present Galileo, a novel Genetic Algorithm to sample fragment spaces. We showcase Galileo in combination with a novel pharmacophore mapping approach, called Phariety, enabling 3D searches in fragment spaces. We estimate the effectiveness of the approach with a small fragment space. Furthermore, we apply Galileo to two pharmacophore searches in the REAL Space, detecting hundreds of compounds fulfilling a HSP90 and a FXIa pharmacophore.

**Supplementary Information:**

The online version contains supplementary material available at 10.1007/s10822-022-00485-y.

## Introduction

The identification of novel leads with a desired pharmacological effect is one of the fundamental challenges in modern drug development. The traditional approach is high-throughput screening (HTS), which is the physical screening of large chemical compound libraries against a target structure. This approach has proven to be ineffective in terms of invested resources compared to acquired hits [1]. An alternative approach is virtual screening, where compound libraries are screened in silico. Only a small number of structures that appear promising are screened physically afterwards. This strategy has proven to increase the success rate and decrease resource investment [2]. In an ideal scenario, the target structure would be compared to an exhaustive enumeration of all possible molecular structures with a certain number of non-hydrogen atoms. However, this approach would require immense computing resources due to the complexity and size of chemical space. Fragment-based drug design (FBDD) was introduced to tackle this problem [3, 4]. In the virtual approach FBDD involves in silico screening of a few small molecules (fragments) against a target structure and combining fragments with high affinity to larger compounds [5, 6]. This approach is fundamentally different from classical (virtual) screening in that one is not limited to an enumerated library of known compounds. On the computational side, combinatorial descriptions of molecules can be utilized to create novel compounds [7].

Fragment spaces are an efficient and elegant way to describe such a virtual, combinatorial library [8, 9]. They consist of a number of fragments with pre-defined attachment points of specific types, so called linker atoms. A set of connection rules defines which linker atoms may be connected to each other. The chemical space spanned by these fragments and rules includes all molecules that may be created by all valid combinations of fragments. Fragment spaces can be created retrosynthetically, i.e. by breaking down larger compounds according to predefined rules [10, 11]. They can also be created in a forward fashion by providing the fragments and combining them with known reactions given by synthesis protocols from literature and lab notebooks [12, 13]. This approach gained a lot of attention in recent years and many pharmaceutical companies created their inhouse fragment spaces in this manner [14]. Enamine’s REAL Space of purchasable make-on-demand compounds is designed in this way, covering more than 29 billion compounds [15].

Due to their combinatorial properties, searching in fragment spaces still poses a difficult challenge [16]. The classical approach of an exhaustive enumeration is only feasible for small fragment spaces or when strongly restricting the possible combinations. Otherwise a combinatorial explosion will occur, making the enumeration infeasible [17]. Moreover, due to their size, fully enumerated spaces not only consume a lot of memory, which would negate the advantages of using a fragment space in the first place. They also are very cost-intensive in terms of computing time and energy consumption. Fortunately, the combinatorial nature of fragment spaces allows for a number of sophisticated search methods. Several methods published in the last two decades have been reviewed recently [18]. Many of these methods combine fragment spaces with pre-existing functionality and—mostly topological—descriptors.

Rarey et al. used tree-shaped descriptors called *Feature Trees* to describe molecular features as a reduced graph. These allowed them to efficiently evaluate the similarity of fragments in a space using a dynamic programming approach [8].

Describing molecules as trees is a common approach. Lauck et al. used a tree-based molecular representation to exhaustively enumerate molecules that fulfill given physicochemical criteria [7].

*DOGS* was developed as a way to automate multi-step synthesis of small moleculular building blocks in silico. *DOGS* first builds initial candidates by exhaustively growing from a number of seed fragments. These initial candidates are then used in another exhaustive growing step during which fragments are added to them until an optimum with respect to a topological similarity value is found, or the molecular weight exceeds a set threshold. It also allows to represent molecules as tree-shaped reduced graphs [19].

Schneider et al. developed *TOPAS*, an evolutionary algorithm that iteratively modifies a selected parent structure that is similar to a query structure with respect to a vector representation of the molecules’ topologies [20].

Fechner et al. employed an evolutionary strategy that modifies molecules by fragmenting them in a retro-synthesis step, which is followed by a synthesis step that recombines compatible fragments. This allows them to generate a broad spectrum of molecules that are similar to a known reference molecule [21].

Ehrlich et al. developed a method which allows them to search fragment spaces for products containing a desired substructure. This is achieved by fragmenting the query structure into subsubstructures which are then matched against the fragments. Compatible fragments that contain adjacent subsubstructures may then be combined into a molecule that contains the desired substructure [22]. This method was extended to allow recursively defined patterns [23].

*Spacelight* is a recent tool which is able to find molecules with optimal topological similarity to a query structure in a few seconds. This requires a preprocessing step in which traditional fragment spaces are converted to so-called *topological fragment spaces*. A query structure is then partitioned in every possible way, resulting in a number of topology graphs. These topology graphs are then used to query the topological fragment space [24].

*SpaceMACS* was developed to find the maximum common induced substructures (MCIS) between a given query molecule and molecules that are encoded in a fragment space without the need to enumerate the products. This is achieved by first matching substructures of the query to fragments. Compatible fragments that extend such a substructure match may then be combined to a larger molecule with an MCIS that bridges multiple fragments [25].

Many of these methods attempt to use classical molecular descriptors with the hope that they behave additively with respect to the combination of fragments. Recent work has shown that this is not necessarily the case and that descriptors like the Connected Subgraph Fingerprint (CSFP), that were specially tailored for fragments and their combination, may perform better and are therefore more desirable for this use-case [26]. As of today, the most frequently applied topological search methods like fingerprint-based similarity, (maximum common) substructure, and reduced graphs can be considered as solved.

While all of these attempts show that the search in fragment spaces is an attractive alternative to virtual screening of enumerated spaces, one type of common task still poses a challenge. Using three-dimensional (3D) information like shape and spatial arrangement of functional groups in the area of fragment spaces proves difficult due to their increased computational cost. Many of the previously mentioned methods only support the integration of 3D descriptors by applying them to enumerated molecules, i.e. products generated by combining fragments. Some noteworthy examples of methods that integrate 3D descriptors directly into the search procedure are FlexNovo [27], Recore- [28], PhDD [29], LigBuilder [30], S4MPLE [31], OpenGrowth [32], the SEED2XR protocol [33], NAOMInext [34] and CONTOUR, which was recently used to successfully discover 11$\beta$-HSD1 inhibitors [35].

The majority of 3D search methods are based on structural alignments, pharmacophore mapping or molecular docking. In this work, we are focusing on pharmacophore models [36]. 3D pharmacophore models have proven to be particularly useful as filters for virtual screening [37, 38]. Due to their abstract character and the limitation to elementary information, hit identification routines are less computationally demanding and can be completed faster. Furthermore, it is even possible to identify structurally diverse hits that still have the potential to bind to the desired target [39, 40]. Pharmacophore modeling techniques are well established in modern drug discovery. The method is discussed in several reviews and their successful application is proven by different examples [41–45].

Previously, Lippert et al. [46] presented Qsearch, a first attempt to integrate 3D pharmacophore queries in fragment space search. Qsearch is based on a simulated annealing approach, which has proven to be capable of finding molecules that fulfill a pharmacophore query in fragment spaces. However, the method was applied only to retrosynthetic fragment spaces with less than 6000 fragments. In addition, Qsearch uses an *undecoration step*, which removes functional groups from hit structures [46]. This leads to structures that are not encoded in the given fragment space, resulting in the loss of knowledge about possible synthesis pathways.

In this paper we present Galileo (**G**enetic **A**lgorithm for **LI**gand and **LE**ad **O**ptimization), a new approach which utilizes a Genetic Algorithm (GA) to search for products encoded in a fragment space optimizing an arbitrary fitness function. GAs are common strategies to solve combinatorial optimization problems in computer science [47]. Previous work has shown that they can be used to efficiently search fragment spaces that were specifically designed for a given application [48, 49]. Galileo is the first GA which directly operates on chemical fragment spaces. As a consequence, hits produced by Galileo operating on the REAL Space can be directly purchased. The most critical aspect of all de novo design methods, namely the quest for easily synthesizable molecules, can therefore be considered as solved.

Additionally, we introduce Phariety, a pharmacophore mapping algorithm which we use as a fitness function within Galileo in an attempt to directly integrate well-established three-dimensional search criteria into the fragment space search. Lastly, we apply Galileo and Phariety to two fragment spaces including the REAL Space, and show that they are able to retrieve hundreds of molecules that are both synthetically accessible and obeying target pharmacophores.

## Methods

Galileo is a standard GA tailored to deal with combinatorial fragment spaces. It implements classical genetic operators like crossover and mutation in a chemistry-aware fashion such that all molecules created are valid fragment space products. The GA can be combined with arbitrary fitness functions. Here, we will demonstrate the functionality of Galileo in the context of pharmacophore searching. In the following, we will first describe the Galileo engine including structure encoding and operators. We then summarize Phariety which roughly follows the backtracking strategy introduced by Kurogi and Gunar [50].

### Genetic algorithm for searching in fragment spaces

**Fig. 1:**
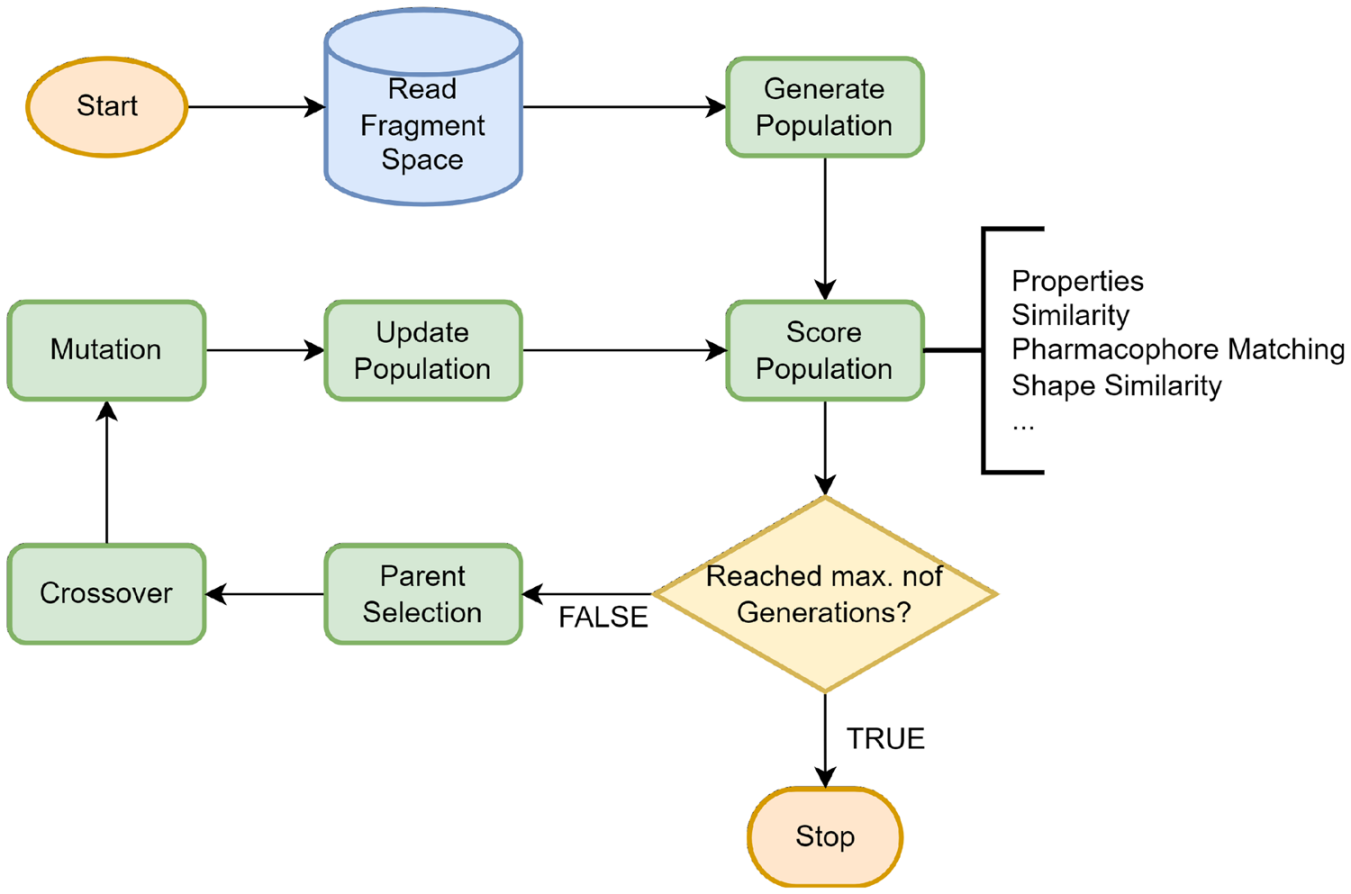
General workflow of Galileo. The fitness calculation is done either via an external fitness function or one of the integrated ones. Phariety has been directly integrated into Galileo

Galileo is a classical GA operating on fragment spaces. A general workflow is shown in Fig. 1. The NAOMI framework [51] is used as the underlying cheminformatics engine.

### Representing fragment combinations

GAs require a representation for valid solutions to an optimization problem called *chromosomes* in this context. Traditionally, bitvectors have been applied for this task. There are several ways one could represent a molecule via a bitvector (e.g. by serializing the covalent bonds). In order to take a molecule’s fragmentation and corresponding connection rules into account, we represent molecules in *chromosomes* as *fragment trees* as described by Lauck et al. [7]: fragments (*genes*) are represented as vertices with potential attachment points that represent the linker atoms. Two vertices are adjacent if and only if the fragments are connected via a bond that is valid with respect to the connection rules. We consider any kind of molecule that may be created by such a combination of fragments a valid solution.

The optimization problem is defined by the scoring or fitness function assigning a numerical score (*fitness*) to each molecule in the solution set. They are described below.

### Initialization

The population is randomly initialized. This is done by creating random fragment trees using the following procedure:

1. pick a random fragment from the fragment space
2. enumerate all linker atoms of this fragment
3. retrieve all fragments from the fragment space that are compatible with at least one linker atom
4. enumerate all fragment/compatible linker combinations
5. select a random combination of fragment and linker atom
6. attach the selected fragment to the corresponding linker atom
7. repeat steps 2–6 considering all fragments in the tree until the tree is saturated (no open attachment points left) or until a user-defined number of fragments is reached

This randomized growing procedure is repeated until the desired population size is reached.

### Crossover

**Fig. 2:**
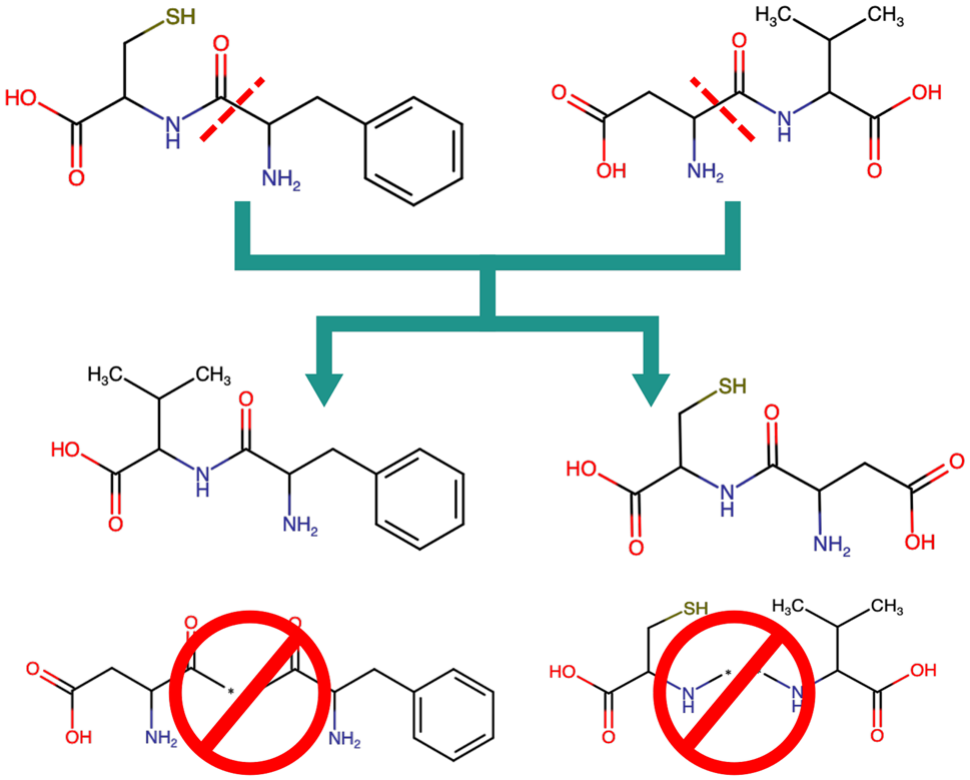
Example of one possible crossover between Asp-Val and Cys-Phe dipeptides. The fragments are split along the peptide bond. Only two of the possible children are valid (Val-Phe and Cys-Asp). The other two children are invalid because the linker atom types are incompatible

Modern fragment spaces often contain a large number of connection rules. Many of these correspond to only a single reaction with a very specific chemical environment around the linker atoms. As such, the number of compatible linker types for any given linker type is orders of magnitude less than the number of linker types. A naive approach to perform a crossover between two chromosomes by randomly picking two chromosomes and randomly picking one edge each in both fragment trees is therefore unlikely to produce any valid offspring. We therefore decided on this alternative approach:

To perform a crossover between two chromosomes, all edges in the first Fragment Tree are enumerated. Each of these edges are considered in turn. The tree is cut along one of these edges. All edges of the second tree that have at least one attachment point that is compatible to either side of the cut are enumerated. For each of these compatible edges, the second tree is cut along that edge. Lastly, all combinations of subtrees of the first and second tree that result in valid Fragment Trees are created, resulting in anywhere between 1 and $4 \cdot n \cdot m$ child trees, where *n* and *m* are the number of edges in the first and second Fragment Tree, respectively (see also Fig. 2). This is repeated for all pair-wise combinations of chromosomes in the selection (see below for the selection methods).

To improve the performance of this step, we use an early-abort strategy where a chromosome combination is only considered if they have at least one compatible linker pair.

### Mutation

**Fig. 3:**
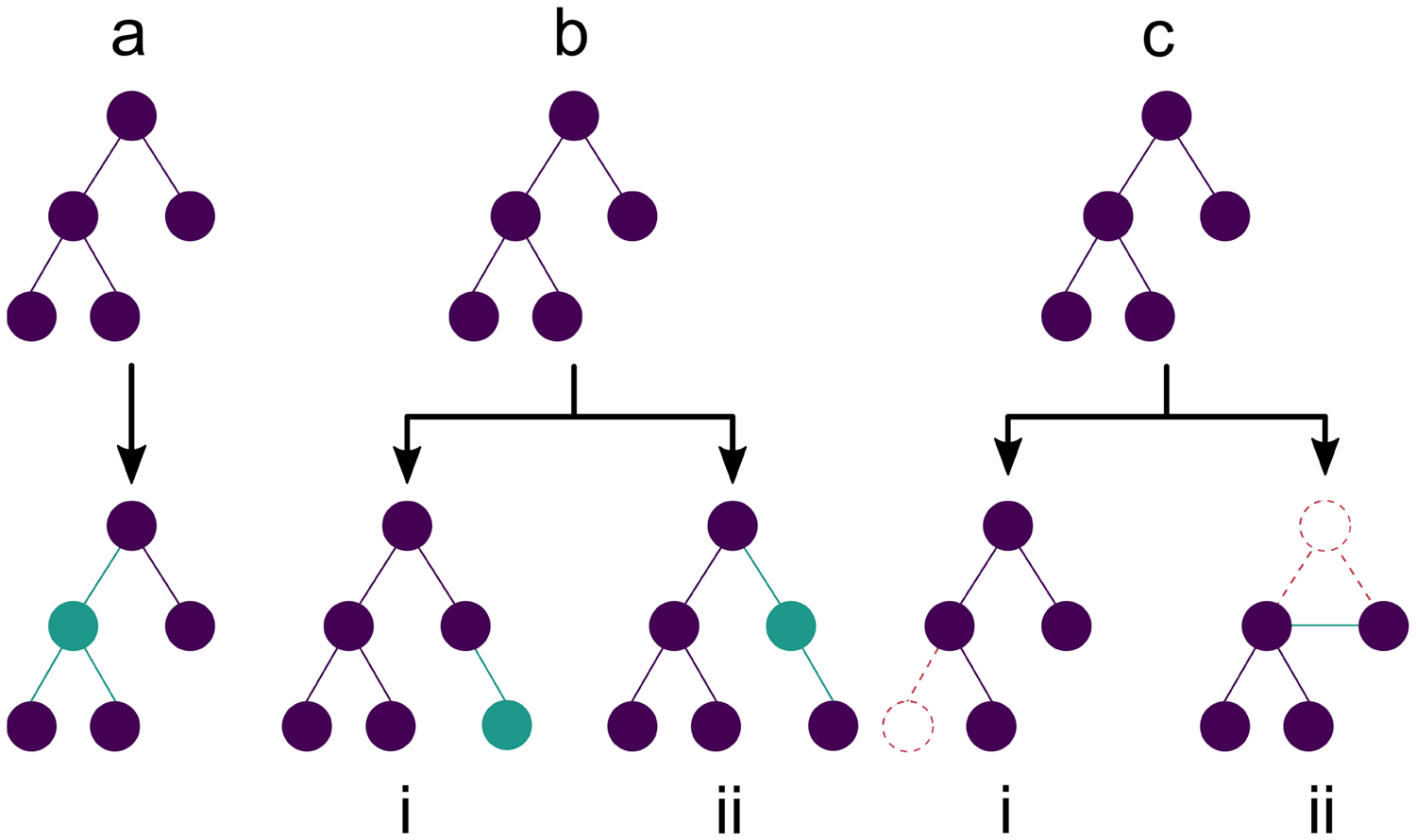
Mutation operations on a fragment tree. **a** Replacement of a fragment. Any fragment within a tree may be replaced; **b i** Addition of a terminal fragment; **b ii** Addition of a non-branching fragment; **c i** Deletion of a terminal fragment; **c ii** Deletion of a non-branching fragment. New fragments and edges are marked in solid green, removed ones are dashed in red

Three mutation operators have been implemented, namely Replacement, Insertion and Deletion. All three modify a single fragment of a fragment tree, i.e. a single vertex or a gene in the GA context. They either replace an arbitrary fragment with a different compatible fragment, add a fragment to a linker atom or in between two other fragments, or remove a fragment with at most two adjacent fragments respectively. In case the removed fragment is connected to two fragments, these fragments must have compatible linker atoms such that they can be connected directly. An overview of the different possibilities is shown in Fig. 3.

### Replacement

Any fragment in a fragment tree may be replaced by another fragment that is compatible to all of the adjacent vertices of the original fragment. The procedure is as follows:

1. Pick a random fragment from the fragment tree and remove it.
2. For each new linker atom, select all fragments from the fragment space with at least one compatible linker atom.
3. Calculate the intersection of all sets obtained in step 2.
4. Filter out all fragments that have fewer linker atoms than the number of surrounding fragments.
5. Remove the original fragment from the candidate set.
6. Assign a probability to each candidate fragment that’s proportional to its similarity to the original fragment.
7. Select a random fragment according to the probability mass function (PMF) from step 6.
8. Consider the selected fragment: Calculate all valid linker atom assignments and pick a random one. If no valid assignment could be found, remove the fragment, adjust the PMF accordingly and go to step 7.
9. Connect the new fragment according to the picked assignment.

Step 3 is required to remove all fragments that can never be a potential candidate because they can’t be connected to all surrounding fragments. Step 4 is required as there may be linker types that are compatible with more than one of the surrounding linker atoms. After having obtained all potential candidate fragments, the valid assignments have to be calculated, i.e a matching between the linker atoms of the candidate fragment and the linker atoms of the surrounding fragments. To calculate these matchings, we build an unweighted bipartite graph. The first set of vertices represents the linker atoms of the candidate fragment:

1 $$\begin{aligned} V_{1} = \{u\ :\ u\ \text {is linker atom of candidate}\} \end{aligned}$$

The second set of vertices represents the linker atoms of the surrounding fragments:

2 $$\begin{aligned} V_{2} = \{v\ :\ v\ \text {is linker atom of surrounding fragment}\} \end{aligned}$$

There is an edge (*u*, *v*) between two vertices if and only if the linker atom types of *u* and *v* are compatible with each other. An assignment for the fragment can then be obtained by calculating a maximum matching *M* on the described graph. The assignment is *valid* if and only if $|M| = |V_1|$. If there is more than one valid assignment, all of them are considered.

The reasoning behind the similarity measurement in step 6 is to prevent the creation of extremely dissimilar compounds via mutation. For this we calculate the Tanimoto coefficient between the CSFP2.5 [26] fingerprints of the candidate fragments and the original fragment. We chose 2 and 5 as the lower and upper bounds because fragments are typically small in size and higher bounds would therefore result in very specific fingerprints. The fingerprints for the fragments are preprocessed and available in a database.

### Insertion

The insertion operator enumerates all linker atoms and edges of a given fragment tree and picks one of them randomly. In case a linker atom was picked, the same growing procedure described above for the Initialization is performed to add a new terminal fragment. If an edge was picked, the tree is split at this edge and a random new fragment that is compatible to both linker atoms is chosen according to the procedure described in the replacement operator.

### Deletion

A fragment may be deleted either if it is a terminal fragment or if it has exactly two adjacent vertices with compatible linker atoms. In the first case, the vertex is simply removed. In the second case, the fragment is removed and the surrounding fragments are connected via the now unused linker atoms.

### Selection

A number of different classical selection methods were implemented, namely: Roulette Wheel Selection (RWS), Stochastic Universal Sampling (SUS), Rank Selection (RS), Tournament Selection (TS) and Random Selection (RAND). The first four have been exhaustively described in previous works [52]. RAND simply chooses a number of chromosomes from the population at random without considering their fitness. The chosen selection method selects a user-defined fraction of the current population which is copied over to the next generation. Afterwards, the offspring generated via the crossover procedure is appended to the new population (see Crossover). The rest of the population is filled with mutations of the Fragment Trees that are already present in the new population. To not completely lose the possibility of further exploration of the solution space, a user-defined fraction of the population is filled with new random fragment trees in the same way as during the initialization.

### Scoring

Galileo provides an interface that allows arbitrary external programs to be used for scoring, as long as they fulfill four criteria: They

1. are scriptable, i.e. they provide a command-line interface
2. take an SDF file as input
3. assign a positive score (incl. 0) to every molecule
4. print the scores in the same order as the input molecules

Additionally, Galileo provides a number of built-in functions that may be used in addition to external programs. This includes the pharmacophore-mapping procedure described later, as well as a number of simple functions that model physicochemical properties. Each property is converted into a score employing one-dimensional Gaussians with a user-defined mean and variance. The supported physicochemical properties are:

- molecular weight
- calculated logP [53]
- number of hydrogen-bond donors/acceptors
- number of nitrogen/oxygen atoms
- number of (aromatic) rings

The built-in functions also include the possibility to define a target molecule and score the population by Tanimoto similarity [54] to this target. As molecular fingerprints, ECFP4 [55] and CSFP2.5 [26] descriptors are available. All internal scoring functions may be combined and weighted. The combined score is calculated by a linear combination of the weighted scores, normalized by the sum of weights.

### Pharmacophore mapping

In order to demonstrate the capabilities of Galileo with respect to 3D searching, we developed and implemented the pharmacophore mapping routine Phariety which is subsequently described. Just as Galileo, Phariety is built on top of the NAOMI framework [51]. The algorithm consists of three main steps, which are discussed in detail in the following paragraphs. In addition to the integration into Galileo, Phariety is available as an independent command line tool for pharmacophore search in virtual compound libraries.

### Preprocessing

The first step of Phariety is a preprocessing step, during which unsuitable molecules are eliminated. In case only a molecule’s topology is given, a set of low-energy conformations can be generated using the tool *Conformator* [56]. Functional groups with specific interaction types are identified for every molecule. To ensure the compatibility with other pharmacophore mapping tools, like Catalyst, Phase and LigandScout [57–59], Greene’s algorithm [60] is used for the identification of hydrophobic interactions. For interaction types like hydrogen-bond donors and acceptors, aromatic interactions, and charged interactions, the NAOMI interaction model is used. The model was derived and extended from the interaction assessment used in FlexX [61]. The assessment is based on a geometric analysis of the overlap of interaction surfaces of two interaction partners and their deviation from optimal interaction geometries. The latter are defined by Rarey et al. [61]. The interaction surfaces of a functional group are used to describe the relative geometric position of possible interaction partners. A topology and geometry analysis of molecular substructures, as described by Bietz [62], is used to assign interaction points to functional groups. Direction vectors are also derived from the analysis of the geometric arrangement of the functional groups. After the assignment of corresponding interaction points to every functional group, a quick compatibility test is performed. Here, number and types of the generated interaction points of a given candidate are tested for their compatibility with the query pharmacophore feature points. The test verifies that the candidate structure contains at least the same number of interaction points of the same type for each interaction type of the query pharmacophore model. Note that the candidate structure may have more features than the query pharmacophore model, which offers the possibility of a partial mapping.

### Mapping algorithm

**Fig. 4:**
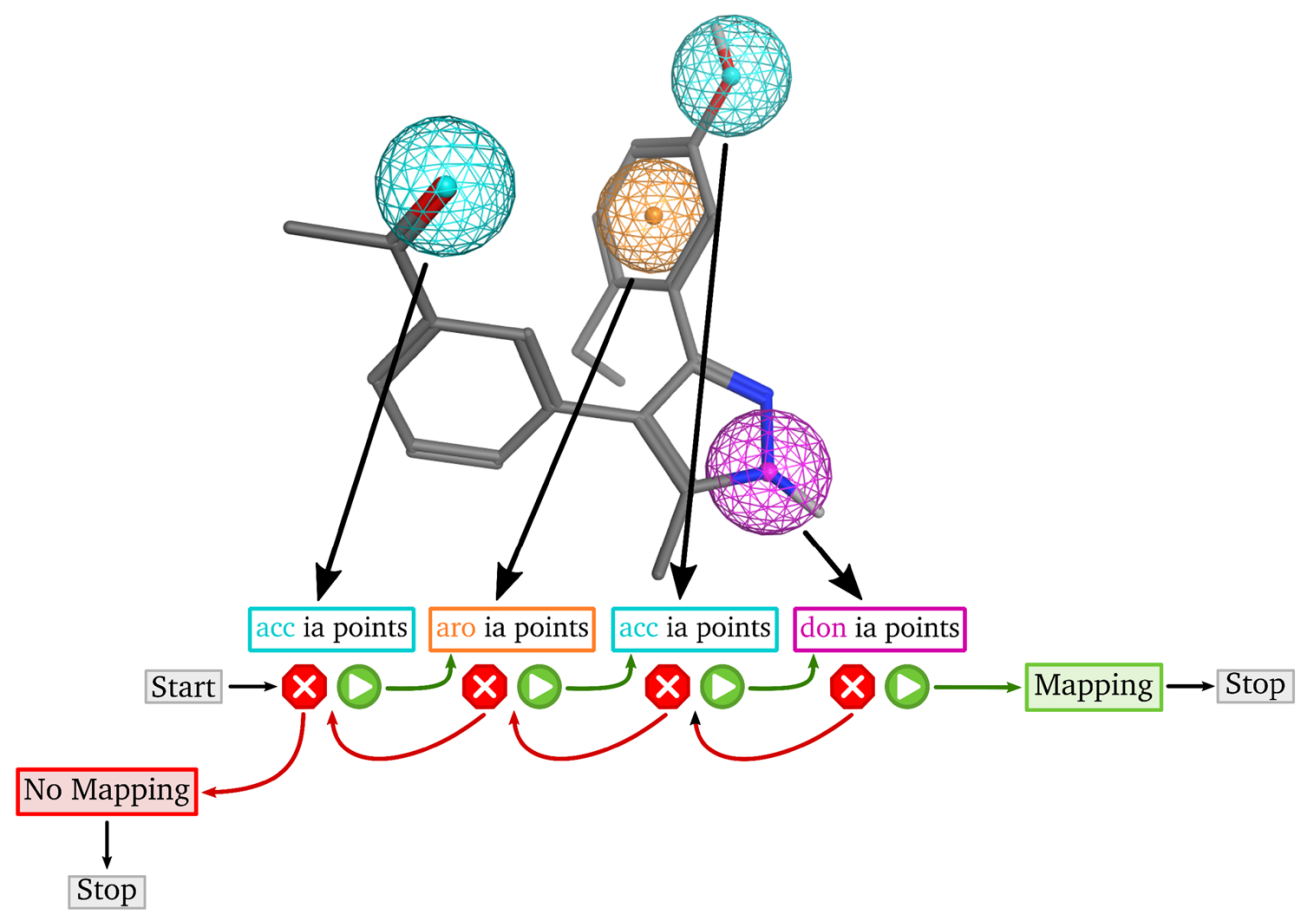
Workflow of Phariety’s mapping algorithm. Query pharmacophore model (top) with underlying ligand structure. *X ia points* stands for all interaction points of the corresponding type of a candidate molecule. The algorithm stops if one valid mapping is found or all possibilities are checked and no mapping can be found

The feature points of the query pharmacophore are mapped onto the interaction points of the candidate molecules using a *depth-first walk with backtracking*, a strategy introduced by Kurogi and Gunar [50]. This greedy algorithm starts with a randomly chosen query pharmacophore feature point and attempts to find a valid compatible interaction point of the candidate molecule. The algorithm places the query pharmacophore feature points in a random order and uses a classical backtracking strategy by assigning compatible interaction points of the candidate molecule one by one. The moment the algorithm runs out of options for a feature point it traces back one step and attempts to find an alternative mapping for the last feature point. The process stops when either a valid mapping is found for every point, or all possible combinations have been tested (see Fig. 4). It is possible to search for either the first or the best mapping. If the latter is chosen, the algorithm continues after the first valid mapping and enumerates all possible remaining combinations. The compatibility test between pharmacophore feature points and candidate molecule interaction points consists of a feature type check and a geometric check. The latter includes the comparison of all distances between the new matched feature point/ interaction point pair and the already mapped points. The deviation between the two distances in the pharmacophore query and the candidate molecule has to be less than or equal to the sum of the two radii of the involved query pharmacophore feature points plus an optional, user-defined tolerance value (see also Fig. A1).

### Postfiltering

A subsequent post-filtering step is performed for all valid assignments. It consists of three tests which verify all constraints of the query pharmacophore model. First, the candidate molecule is transformed onto the query pharmacophore model using Kabsch’s algorithm [63, 64]. Afterwards, the first test checks whether the centers of the interaction points are localized within the spheres of the corresponding pharmacophore feature points, while allowing a user-defined deviation (see Mapping Algorithm above). The second test verifies whether potentially defined directions of query pharmacophore points are compatible with the corresponding, mapped interactions of the candidate molecule. The angle between the corresponding directions has to be less than a user-defined threshold. A special case are terminal and rotatable acceptor and donor interactions points. Those interaction points are rotated onto the query feature points before checking the angular constraint. Note that this may cause a minor deviation in the hydrogen coordinates of the returned conformation from the original. The third test verifies that the heavy atoms of the transformed candidate molecule do not overlap with exclusion volume spheres if any are defined in the query pharmacophore. The van-der-Waals radii of the heavy atoms are used for this overlap test. A partial overlap may be allowed by the user.

### Score calculation

A score in the range of [0, 1] is calculated for mappings that pass all postfiltering steps, where 1 means an ideal mapping without deviation, and 0 means that no valid mapping is possible. The latter is only returned if one of the checks in the postfiltering step fails or no mapping can be found in the first place. The score is a weighted linear combination of two normalized terms: the distance deviation ($s_{distance}$) and the direction angle deviation ($s_{direction}$):

Let $i \mapsto m(i)$ be the mapping of the query feature point *i*.

3 $$\begin{aligned} s_{distance} = \frac{1}{\sum _{(i,j)} (r_i + r_j + \epsilon )} \cdot \sum _{(i,j)} |d_{(i,j)} - d_{(m(i), m(j))}| \end{aligned}$$

where $r_i$ and $r_j$ are the radii of query feature points *i* and *j*, $d_{(i,j)}$ is the distance between the query feature points *i*, *j* and $d_{m(i), m(j)}$ is the distance between the mapped interaction points *m*(*i*), *m*(*j*) of the candidate molecule.

4 $$\begin{aligned} s_{direction} = \frac{1}{\gamma \cdot n} \cdot \sum _{i} \angle (v_i, v_{m(i)}) \end{aligned}$$

where *n* is the total number of regarded angles between direction vectors, $\angle (v_i, v_{m(i)})$ is the smaller angle between the direction vector $v_i$ of the query feature point *i* and the direction vector $v_{m(i)}$ of its mapped interaction point *m*(*i*) of the target.

The weights are user-defined and must add up to 1.0. The weight of the angle deviation term is set to 0.0 if no direction information exists. Per default, both weights are set to 0.5. The final score is given by:

5 $$\begin{aligned} s_{total} = 1 - (w_{dis} \cdot s_{distance} + w_{dir} \cdot s_{direction}) \end{aligned}$$

where $w_{dis}$ is the weight for the distance deviation term and $w_{dir}$ is the weight for the angle deviation term.

The algorithm returns a score for each candidate molecule and, if possible, the corresponding conformation superposed onto the query pharmacophore model.

## Results

For our validation and experiments we used a version of the REAL Space which encodes an estimated $19\times 10^9$ molecules [13]. This fragment space contains fragments that are based on commercially available substances and synthesis protocols by Enamine Ltd. Additionally, we used a smaller fragment space which we call *ZB SampleSpace*. The space consists of two sets of educts, all commercially available from Sigma-Aldrich [65], combined with the software CoLibri [66]. The first set contains 12,541 reactants with a molecular weight in the range of 250 to 400 Dalton. The reactants from this list represent late-stage intermediates for which a number of synthesis steps have already been performed. The second set of 2205 reactants contain molecules with significantly more functional groups and lower molecular weight, typically used for final derivatization. The molecular weight of these reactants are in a range of 10 up to 175 Dalton. The functional groups that occur in the sets are shown in the Supplementary Information (Tables A1 and A2). The standard set of 120 reactions of CoLibri was used for space generation. For further information on the generation of chemical fragment spaces see [12, 66]. A fully enumerated version of this space contains 11,178,764 molecules.

### Phariety benchmark

To evaluate the performance of Phariety, we recreated the experiment of Spitzer et al. [67]. We rebuilt two pharmacophore models of two targets of the maximum unbiased validation (MUV) dataset [68]: heat shock protein 90 (HSP90, PDB: 2BT0 [69]) and factor XIa (FXIa, PDB: 2FDA [70]). Heat shock protein 90 is a molecular chaperone, which takes part in the folding, activation and stabilization of proteins. The activated form of factor XI is a serine protease involved in the blood coagulation cascade. For further details on the targets we refer to Spitzer et al. [67] As stated by Spitzer et al., the MUV data set was chosen due to three different reasons. First, it contains only known active and experimentally verified inactive compounds for each target. Furthermore, the given inactive compounds are structurally close to the active compounds, whereby artificial enrichment should be prevented. Lastly, the size of the data set is convenient for the given application. Both targets were chosen due to the existence of comprehensive X-ray data including complexes with drug-like ligands. Moreover, the interactions between ligand and protein allowed Spitzer et al. to create query pharmacophore models with only a few hydrophobic features. As concluded by Wolber et al. [71], the interpretation of hydrophobic features differs greatly between different pharmacophore mapping algorithms. Therefore, Spitzer et al. tried to focus on more comparable features, like hydrogen-bond features [67]. We used Phariety and the pharmacophore module of the Chemical Computing Group’s Molecular Operating Environment (MOE) [72] for the recreation of the *unmodified* pharmacophore queries of Spitzer et al. The queries were derived directly from their protein-ligand complex structures taken from the PDB. Both queries consist of one hydrogen-bond donor, two hydrogen-bond acceptors and one hydrophobic feature point. As expected, MOE and our pharmacophore generation routine place the hydrophobic features slightly differently. The remaining features are placed at the same coordinates. The resulting models are shown in the Supplementary Information (see Figs. A2–A5). The hydrophobic feature of FXIa has a radius of 1.5 Å. The hydrophobic feature of HSP90 has a radius of 2.0 Å and the hydrogen bond feature has a radius of 1.25 Å. All remaining features have a radius of 1.0 Å.

Following the example of Spitzer et al. [67], no information about directions of the hydrogen bond features were included in this initial experiment. However, each query includes information about the exclusion volume. The exclusion volume spheres were placed around each heavy atom of the corresponding protein’s binding site using MOE. This resulted in 324 spheres for FXIa and 353 for HSP90 respectively. We used the default generated radii of MOE for the exclusion volumes. These ranged from 1.3 to 1.95 Å for both queries. In Phariety, the van-der-Waals radii of ligand heavy atoms are not allowed to clash with the exclusion volume spheres. However, MOE only regards the center of the ligand heavy atoms and does not take their van-der-Waals radii into account. For comparison purposes, we extended Phariety accordingly. For each query model, a multi-conformer screening library was generated from the active as well as the inactive compounds of the MUV data set using Conformator [56]. Starting with the 30 known active compounds and 15,000 decoy compounds for each target, we ended with 4177 active and 2,735,282 inactive conformations for HSP90, and 5178 active and 2,750,353 inactive conformations for FXIa respectively. We used the generated multi-conformer libraries for both applications. Together with the described models we started the applications with default settings and compared the resulting hit lists.

As concluded by Spitzer et al., different pharmacophore hit retrieval algorithms and different chemical feature definitions lead to differences in the resulting hit lists. We confirmed this regarding MOE and Phariety, as shown in Table 1 (see also Fig. A6). Nevertheless, both algorithms retrieve a similar amount of hits, which also corresponds with the results of Spitzer et al. The high proportion of common hits demonstrates that the performance of Phariety is in line with widely used pharmacophore search tools.

**Table 1:** Results of the benchmark experiment of phariety and MOE

	HSP90		FXIa	
	Active	Decoys	Actives	Decoys
MOE	5	1958	2	715
Phariety	6	1751	5	1024
Common hits	4	1207	2	447

The displayed numbers are recovered molecules of the corresponding dataset, not conformers

To roughly evaluate the runtime requirements, the process time for searching 2,750,353 small molecules against the FXIa target was measured. Phariety needed on average 0.032 ms per conformation. In comparison, the runtime of MOE is in the same ballpark with 0.047 ms per conformation. For both applications we did not include the time for preprocessing the conformations, which includes loading of the structures and generation of the pharmacophore points. The run time analysis was performed on an Intel Core i5–8500 processor with six 3.00 GHz cores.

### Galileo validation

To validate Galileo, we first performed a proof of concept experiment in which we want to compare the performance of Galileo with that of random sampling. For this experiment we chose three well-known drugs: Rivaroxaban (an anticoagulant), Imatinib (cancer medication) and Lopinavir (an HIV protease inhibitor). We attempted to find similar compounds that are encoded in the REAL Space. For the fitness function, we evaluated the Tanimoto similarity of the CSFP2.5 fingerprints between the respective target and the population molecules. We set the population size to 10,000. To evaluate the influence of the genetic operators on the population, we increased the number of generations of the GA for every run by one, starting at 1, up to a maximum of 50. We executed Galileo 10 times for each target structure and generation number. Afterwards, we evaluated the best fitting molecule, as well as the mean and minimum of the top 50 best fitting molecules after removing duplicates.

We compared these results to a random sampling of the fragment space. This was done by setting the number of generations for Galileo to 0, which effectively is equal to a random sampling of the fragment space. To accommodate for the potential random sampling included in the advancement of a generation, the population size for the random sampling was increased proportionally to the number of generations in Galileo. This way, the total number of molecules that we encounter during the execution of the GA and the random sampling was equal.

**Fig. 5:**
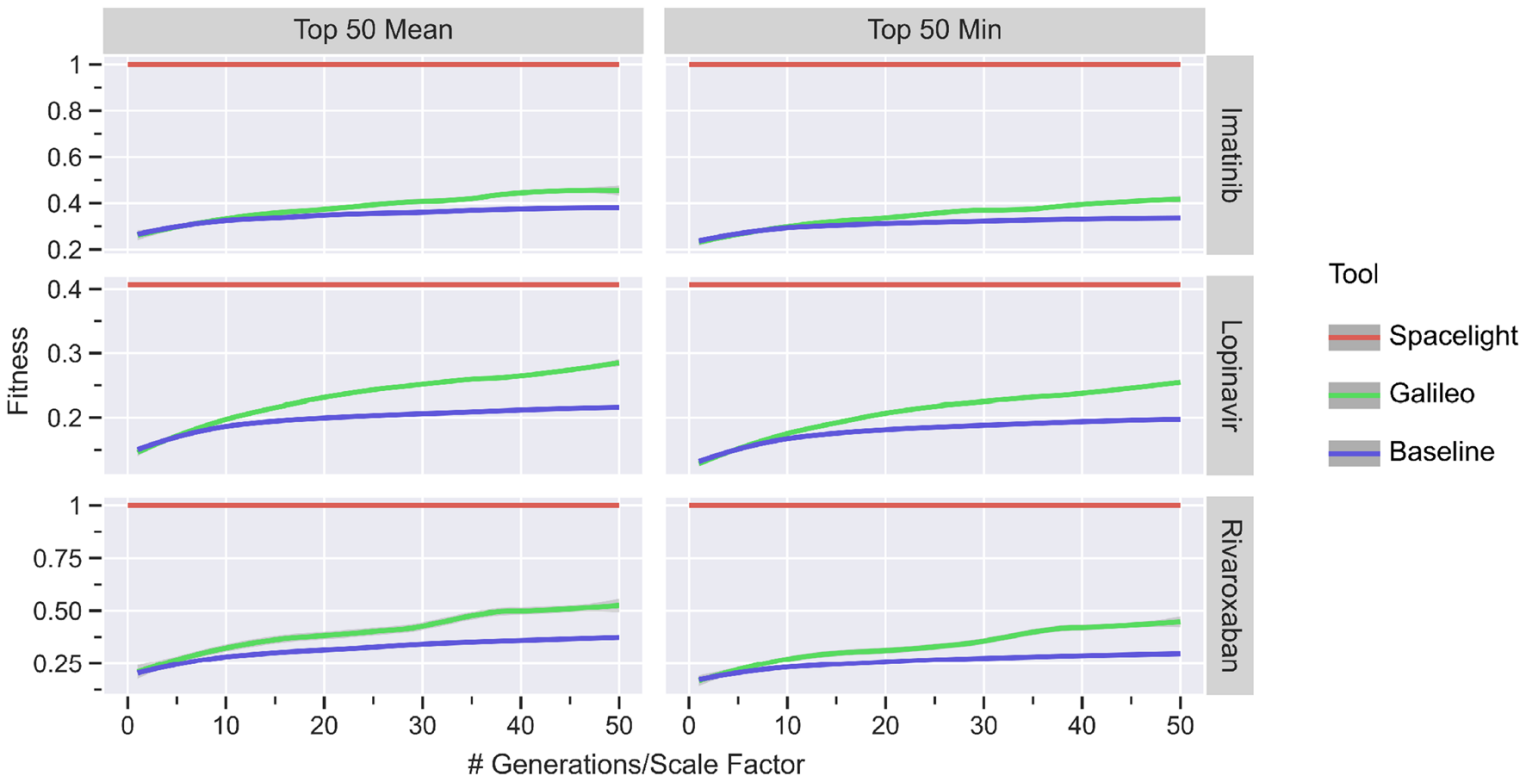
Results for the Galileo validation experiment. The mean and minimum fitness of the top 50 molecules (after removing duplicates) observed in the random sampling and last generation of the GA are shown. The smoothed lines and confidence intervals are generated via LOESS over 10 runs for each number of generations

To figure out the best possible similarity value, we additionally converted the REAL Space to a topological fragment space and used the three target structures as queries for SpaceLight [24]. The combined results can be seen in Fig. 5. The raw output data for all of these experiments, including used computational resources and elapsed time, is available in the Supplementary Information.

We expected the GA and the random sampling to perform very similarly on average for a low number of generations. With an increasing number of generations, we expected to see the GA outperform the random sampling due to the nature of the GA—i.e. prefering fitter molecules during selection and modfying these fitter molecules to slowly accumulate more and more molecules with a high fitness value. The results show that on average, this is indeed the case. For the top 50 mean and minimum, the GA significantly outperforms random sampling after a critical number of generations for all three target molecules.

We conclude that Galileo may be considered a valid alternative to naive enumeration of a fragment space, even for easy to calculate fitness functions.

### Using Galileo with Phariety

**Fig. 6:**
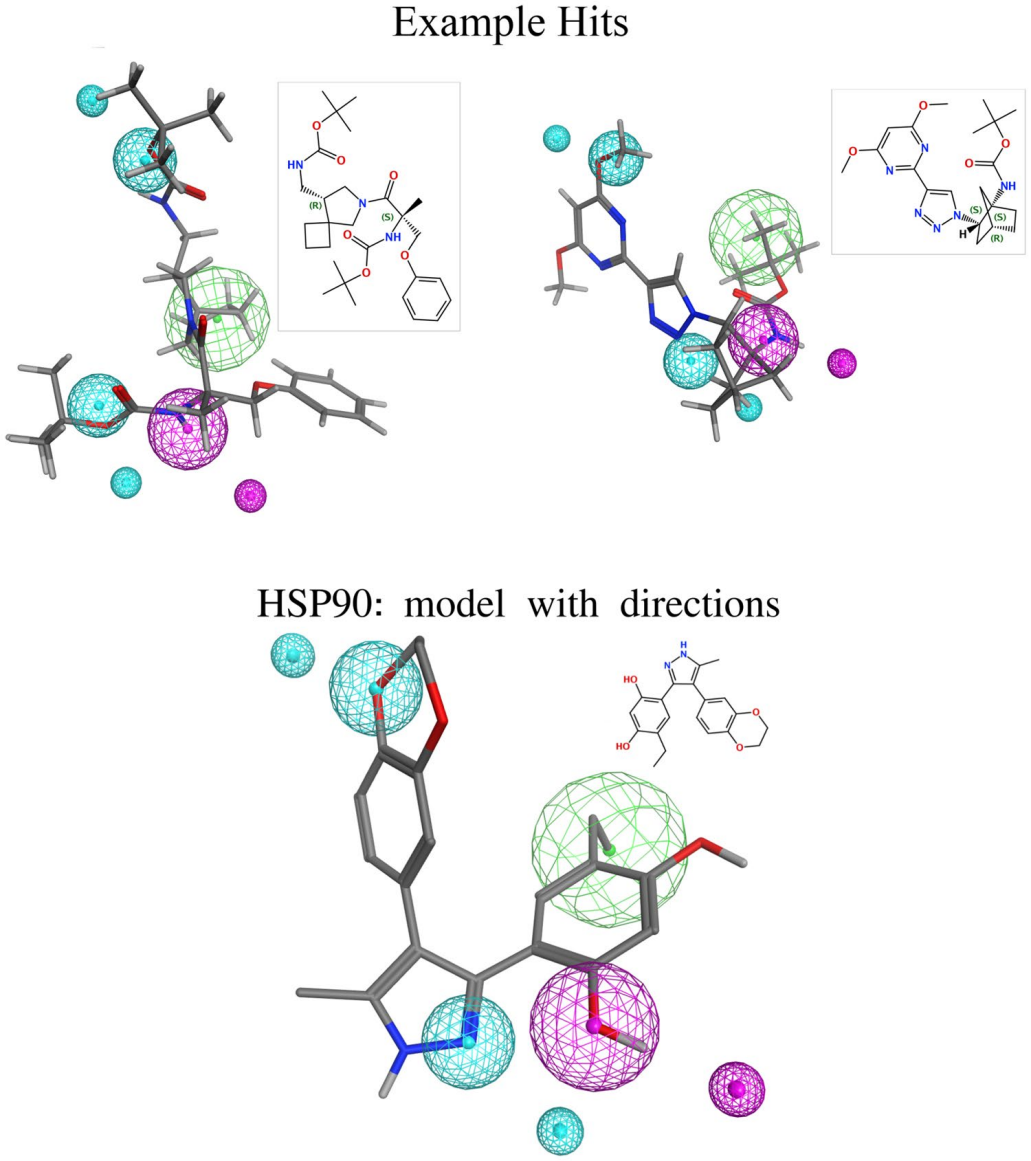
Query pharmacophore model for HSP90 (bottom) and two examples from the hit list of the REAL Space (top). Direction information are displayed as smaller spheres in the same color as their corresponding feature. The hydrogen bond acceptor features are displayed in cyan, the hydrogen bond donor features in magenta and the hydrophobic features in green. The query pharmacophore model is superposed and visualized onto both example hits. The example hit conformers were generated after the sampling using Phariety. All 3D representations and structure diagrams are generated using MOE

To show the performance of Galileo in 3D searches, we first performed a pharmacophore search with the HSP90 and FXIa pharmacophores on the fully enumerated ZB SampleSpace using Phariety. For this experiment we did not include exclusion volumes, and included direction information for the hydrogen-bond features. The direction vectors as generated by our interaction model are shown as smaller spheres in Figs. 6 (bottom) and 7 (bottom).

**Fig. 7:**
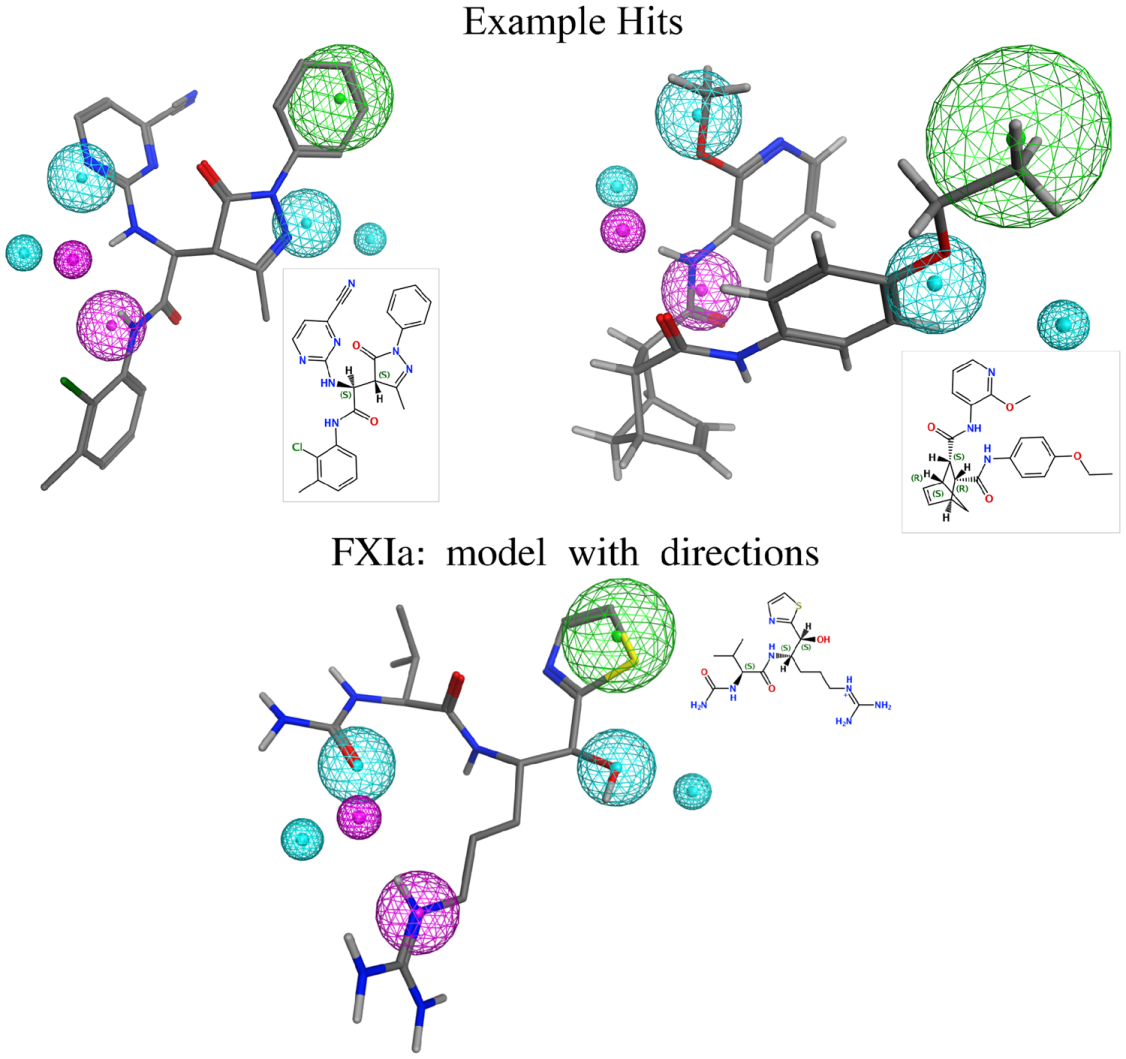
Query pharmacophore model for FXIa (bottom) and two examples from the hit list of the REAL Space (top). Direction information are displayed as smaller spheres in the same color as their corresponding feature. The hydrogen bond acceptor features are displayed in cyan, the hydrogen bond donor features in magenta and the hydrophobic features in green. The query pharmacophore model is superposed and visualized onto both example hits. The example hit conformers were generated after the sampling using Phariety. All 3D representations and structure diagrams are generated using MOE

After this initial search, we executed Galileo with the ZB SampleSpace, the same pharmacophores, and Phariety as the fitness function. We chose 20 and 20,000 as the number of generations and population size respectively. The experiment was repeated 5 times. Additionally, we randomly sampled $4\times 10^5$ products from the fragment space as a baseline. This was also repeated 5 times. Phariety was set up with default parameters, a single conformer for each molecule and an allowed angle deviation of 40 degrees.

Out of a total of 452 hits for FXIa, Galileo was able to retrieve 55 hits ($\approx 12\%$) across all five runs. 14 unique hits were recovered during the best run. The average hit rate was $2.7\%$. In contrast, the random sampling recovered 46 unique hits across the five runs. The average hit rate in the final population of Galileo and the random sampling can be seen in Fig. A7, top-right.

We repeated the same experimental setup with the HSP90 pharmacophore. This time however, we left out the directional constraints because an inspection of the enumerated space revealed that only two of the products match the pharmacophore with directional constraints. With this modified pharmacophore, a total of 247,251 hits were found in the enumerated ZB SampleSpace using Phariety ($\approx 2.2\%$ of the space). Out of these, Galileo recovered 19,207 unique hits across all five runs ($\approx 7.8\%$). Meanwhile, random sampling recovered 40,187 hits ($\approx 16.3\%$). The average hit rates in the last population can be seen in Fig. A7, bottom-right.

While the results for the HSP90 runs might seem unexpected at first, one has to keep in mind that the used pharmacophore is very unspecific. As a consequence, a lot of structures match this query and the probability that the random sampling finds a match is high. As an aside, Galileo is designed to optimize the initial structures towards the query and not return a broad selection of compounds. While a search with an unspecific query is possible, it does not fit the the expected application scenarios of Galileo. Users might want to consider different approaches for such scenarios.

Lastly, we let Galileo optimize products from the REAL Space. The experimental setup was the same as before, with the exception that the population size was increased by a factor of 10 for both Galileo and the random sampling to accommodate for the increase in the size of the fragment space. We also used the unmodified version of the HSP90 pharmacophore without exclusion volume and with directional constraints. Since a full enumeration of the REAL Space is nearly impossible, we only compared the results to the baseline, i.e. random sampling of the fragment space. For FXIa, a total of 820 unique hits were retrieved by Galileo with an average of approximately 193 unique hits per run. The total mean score was approximately 0.78 (median $\approx 0.80$). For HSP90, Galileo found a total of 17 hits with an mean and median score of approximately 0.70. The average hit number across the runs was 3.4. In comparison, the random sampling found 380 and 17 hits for FXIa and HSP90 respectively. The average hit number in the final population can be seen in Fig. A7, left.

**Fig. 8:**
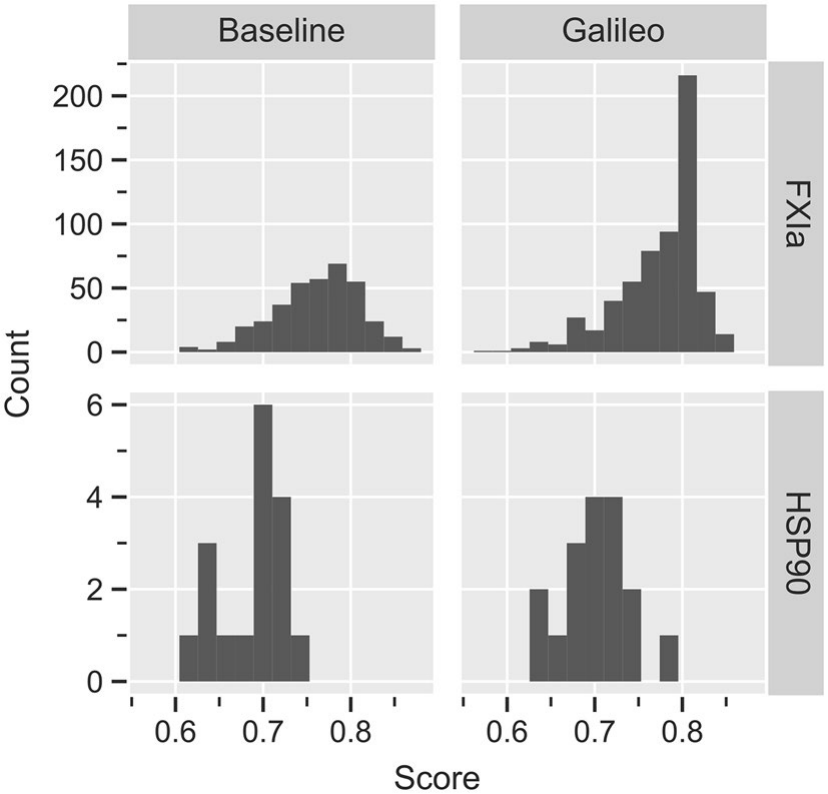
Fitness distribution of molecules generated by Galileo and random sampling. Molecules are taken from the final population of Galileo and the random sampling after sampling the REAL Space and dropping duplicates

**Fig. 9:**
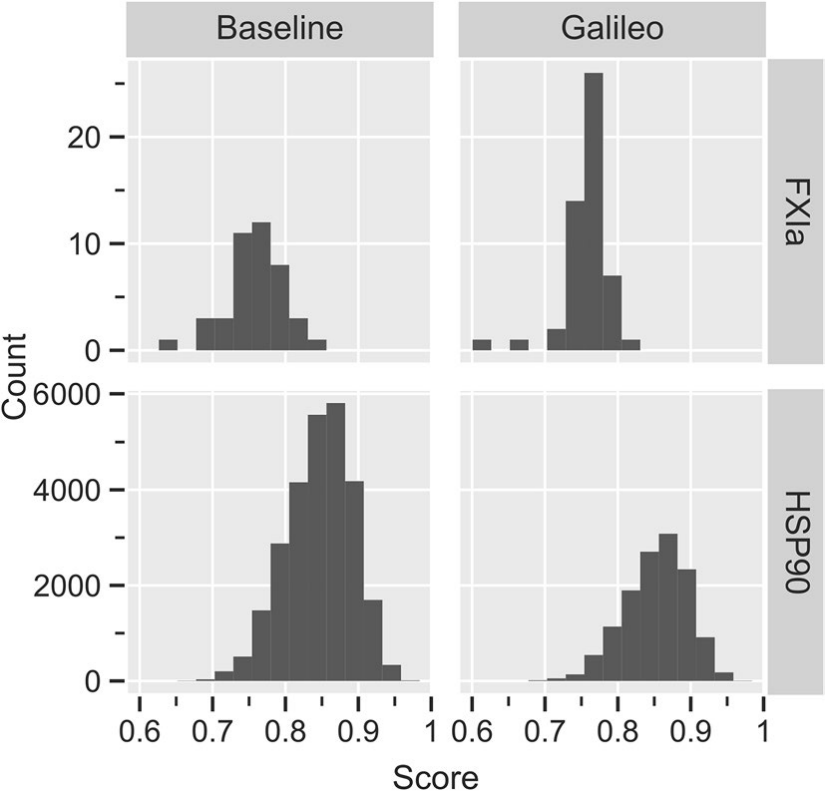
Fitness distribution of molecules generated by Galileo and random sampling. Molecules are taken from the final population of Galileo and the random sampling after sampling the ZB SampleSpace and dropping duplicates

The distribution of scores for both experiments can be seen in Figs. 8 and 9.

A selection of the hits for HSP90 and FXIa from the REAL Space can be seen in Figs. 6 and 7 respectively. We expected the hits to mostly fulfill the pharmacophore model, while having a low structural similarity to the original ligand structure. All interaction points were indeed occupied by corresponding functional groups. However, the directionality of hydrogen bond acceptors and donors was often not satisfactory. This highlights that the balance between the distance term and the direction term is important to obtain a reasonable score.

## Discussion

Pharmacophore searching is one of the key technologies for ligand-based virtual screening. So far, most available methods were only able to screen existing libraries. Here, we presented a novel approach based on a GA which enables to search for molecules obeying a pharmacophore in a combinatorial fragment space for the first time.

In large spaces like the REAL Space, Galileo is the first 3D geometric search engine. Even for small spaces, the approach demonstrates its substantial run time advantage: Searching the fully enumerated ZB SampleSpace with the pharmacophore mapping routine took approximately 1 week for each pharmacophore, whereas each run of Galileo for this space was completed in just 2 days for FXIa and 1 day for HSP90. The random sampling took approximately 3 days to complete. A big advantage of Galileo is that it decouples the search process from the fitness function. In this paper, we demonstrated the performance of Galileo on pharmacophore searching, however other 3D search methods like structural alignments or even molecular docking can be used with a reasonable amount of computing power. Even for cases in which the fitness function is simple or unspecific (e.g. fingerprint similarity or pharmacophores without directional constraints), Galileo is still preferable over exhaustive enumeration or random sampling because the number of molecules that have to be held in memory or written to disk is significantly lower, the quality of the acquired hits (i.e. their score) is higher, and the runtime is significantly lower (see Figs. A8–A13).

The decoupling of the fitness function from the search process comes with well-known disadvantages. First of all, GAs are randomized and heuristic. Therefore, a performance guarantee of any kind cannot be given. Second, specific properties of the fitness function might not be usable. Galileo in combination with Phariety, for example, cannot make use of the very restrictive exclusion volumes usually being part of pharmacophore models. This is due to the fact that it is very unlikely that the GA manages to create products from fragments that both match the pharmacophore and don’t clash with the exclusion volume at the same time.

So far, Galileo doesn’t create conformers and depends on the scoring function to create them if required. This also means that any 3D information is lost upon exiting the scoring function and doesn’t carry over to the next generation. Upon execution of a mutation or crossover operation, the fragment tree changes and a new conformation would have to be generated either way due to potential clashes created by naive connection of two 3D-fragments. Galileo remedies this issue somewhat by caching scores for products that have been encountered before.

Additionally, Galileo currently does not evaluate the fitness of the individual fragments that make up a molecule. This is due to the fact that we designed Galileo to be as generic as possible. However, it may be interesting to evaluate the fitness of fragments for a more directed optimization. This is something we wish to address in the future.

In summary, Galileo is a starting point for 3D searching in fragment spaces. We believe that it is already of high value for drug design projects building on fragment spaces like the REAL Space. Since highly effective and efficient algorithms have been developed for topological searching in fragment spaces [24, 73], Galileo should be considered as the baseline system for better algorithms to be developed in the future.

## Supplementary Information

Below is the link to the electronic supplementary material.

Supplementary file1 (ZIP 244732 kb)

Supplementary file 2

Supplementary file 3 Supplementary file 3a Supplementary file 3b

Supplementary file 4 (PDF 31165 kb)

## Data Availability

All data generated or analysed during this study are included in this published article and its supplementary information files.
