# Supplementary material for: Galileo: Three-dimensional searching in large combinatorial fragment spaces on the example of pharmacophores
Source: J Comput Aided Mol Des. 2022 Nov 24;37(1):1–16. doi: 10.1007/s10822-022-00485-y (PMC10032335; doi:10.1007/s10822-022-00485-y)
Supplement: Supplementary file 6 — Supplementary file 4 (PDF 31165 kb) [file 10822_2022_485_MOESM4_ESM.pdf]

# Appendix A Supplementary Information

**Table A1 Percentages of the functional groups as they occur in the molecules of the educt set 1 of the *ZB SampleSpace*.** Allowed elements are `C, H, N, O, S, F, Cl, Br, I, B`

| Functional group | Percentage |
|---|---|
| Alcohols | 14.1 |
| Amines | 20.3 |
| Carboxylic acids | 15.2 |
| Halides | 50.4 |

**Table A2 Percentages of the functional groups as they occur in the molecules of the educt set 2 of the *ZB SampleSpace*.** Allowed elements are `C, H, N, O, S, F, Cl, Br, I, B`

| Functional group | Percentage |
|---|---|
| Acid chlorides | 3.1 |
| Alcohols | 1.1 |
| Aldehydes | 8.2 |
| Amidines | 2.2 |
| Amines | 1.8 |
| Boronic acids | 11.2 |
| Carboxylic acids | 18.0 |
| Chloroformiats | 0.5 |
| Halides | 16.6 |
| Hydrazines | 0.7 |
| Isocyanates | 2.5 |
| Ketones | 0.3 |
| Sulfonylchlorides | 2.4 |

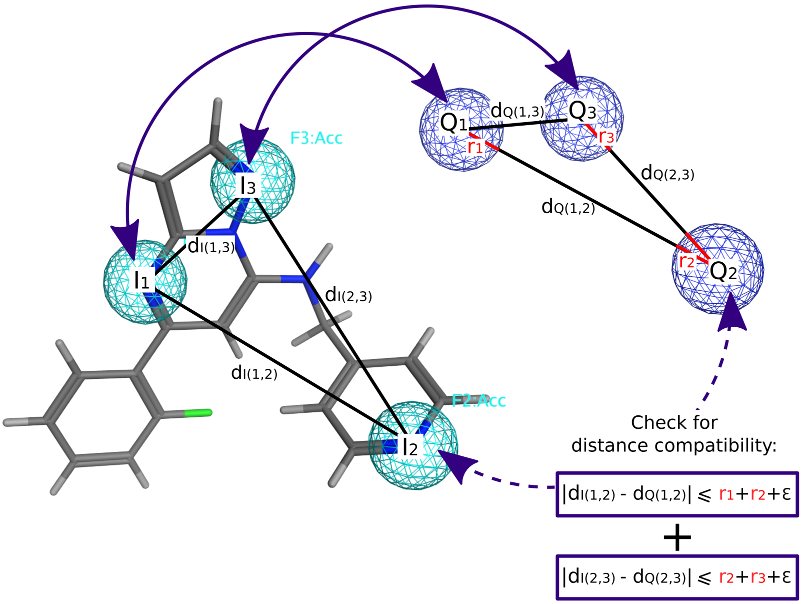


**Fig. A1** Distance compaitiblity measure that Phariety is using to ensure valid mappings. A mapping is valid if the pairwise distances between the point centers are within a certain threshold given by the distance and the radii of the query pharmacophore points. If the distance exceeds the sum of both radii and a user-defined allowance, the mapping is invalid

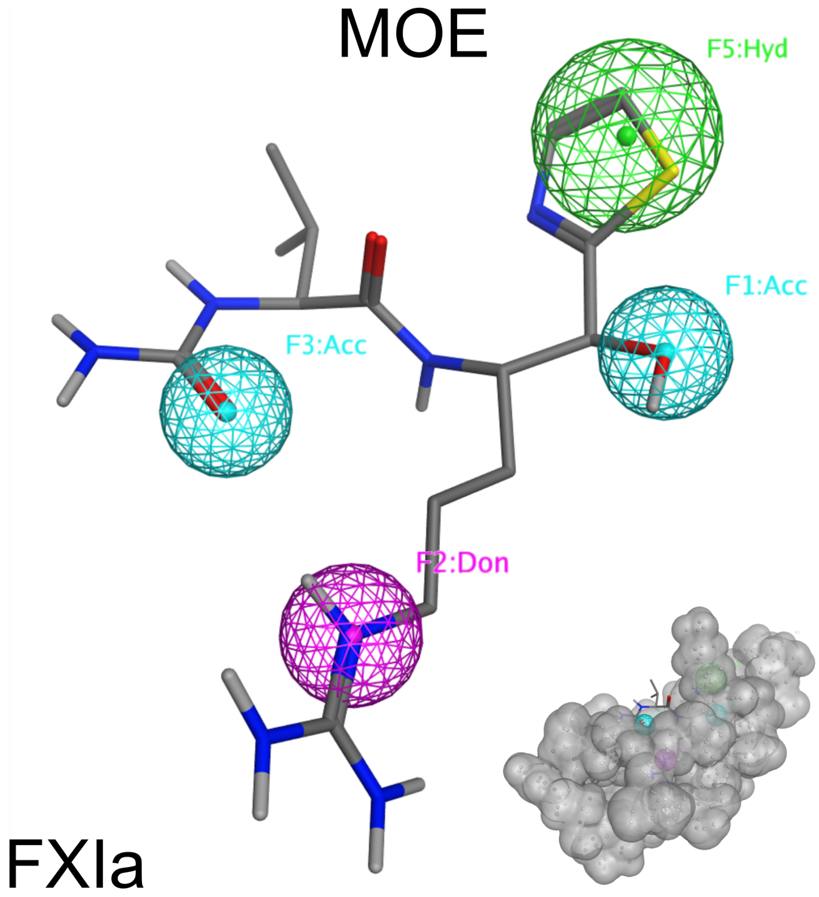


**Fig. A2** Pharmacophore model for FXIa as automatically generated by MOE. The exclusion volume is shown in the bottom right corner

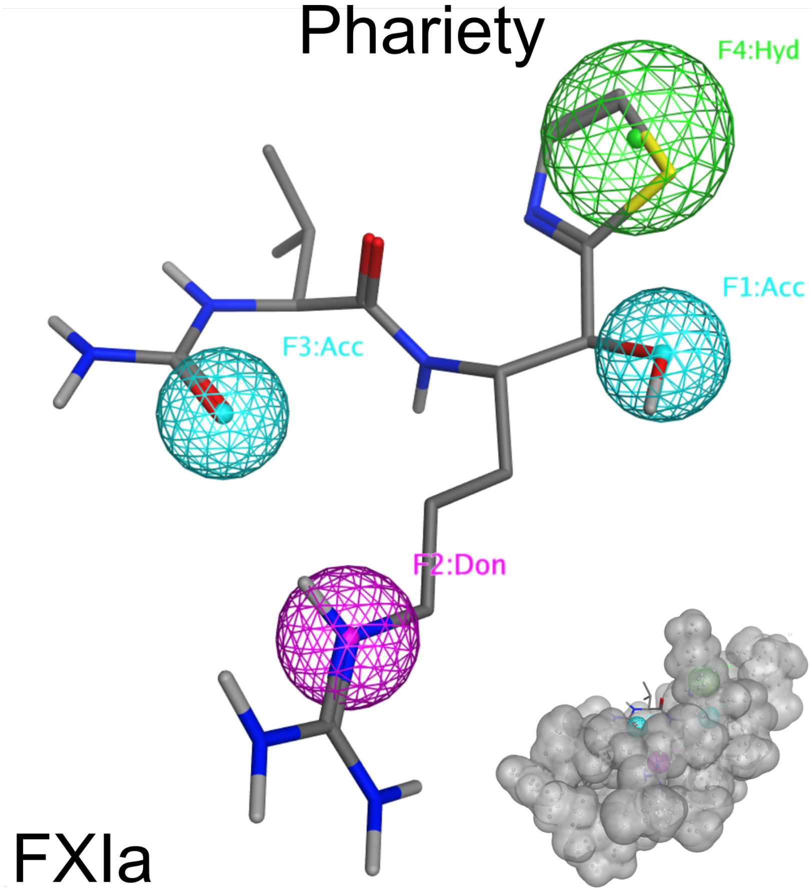


**Fig. A3** Pharmacophore model for FXIa as automatically generated by Phariety. The exclusion volume is shown in the bottom right corner

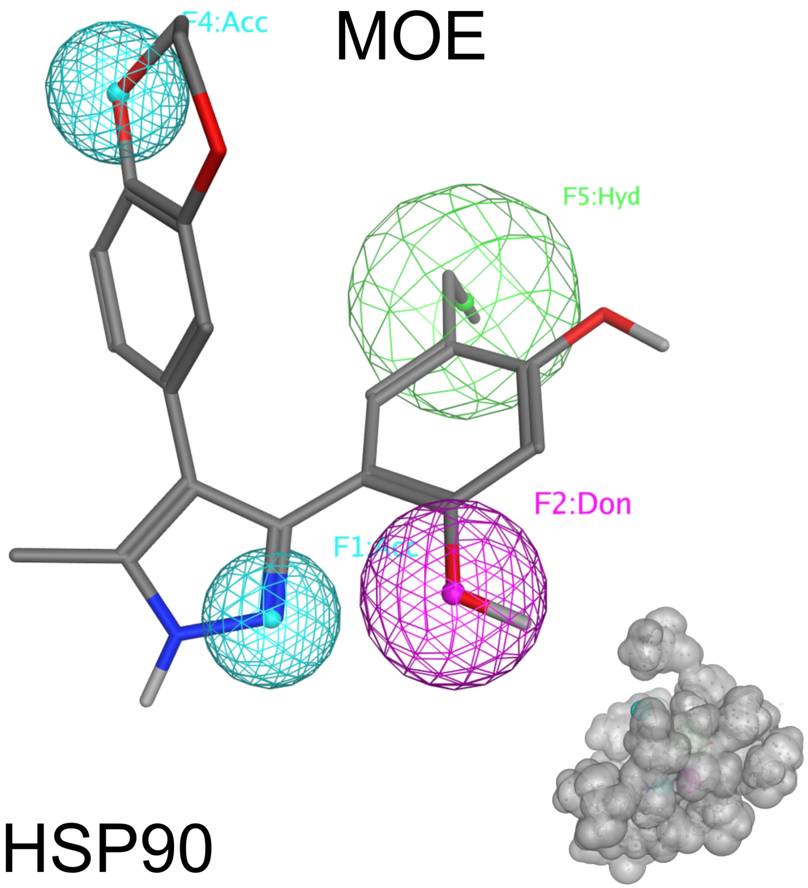


**Fig. A4** Pharmacophore model for HSP90 as automatically generated by MOE. The exclusion volume is shown in the bottom right corner

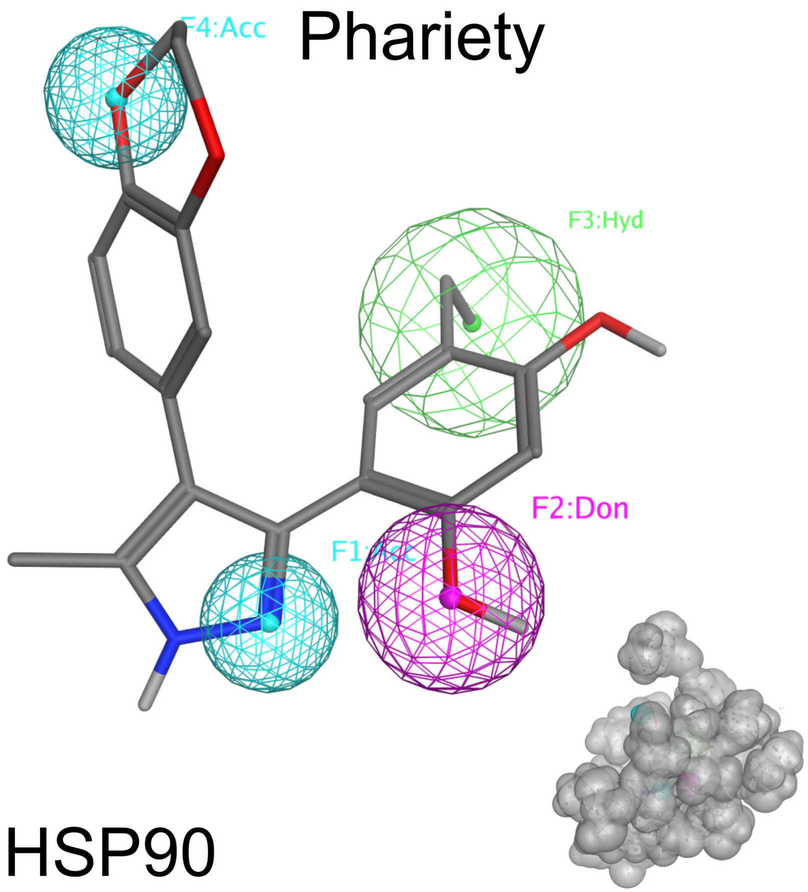


**Fig. A5** Pharmacophore model for HSP90 as automatically generated by Phariety. The exclusion volume is shown in the bottom right corner

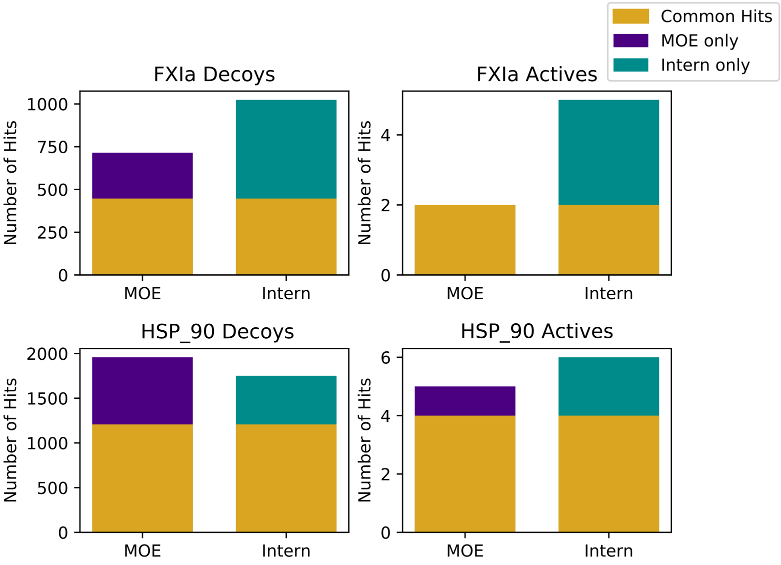


**Fig. A6** Comparison of the pharmacophore-mapping performance of MOE and Phariety. FXIa and HSP90 actives and decoys were mapped to the corresponding pharmacophore. Yellow hits were found by both tools. Purple hits were recovered only by MOE. Turquoise hits were recovered only by Phariety

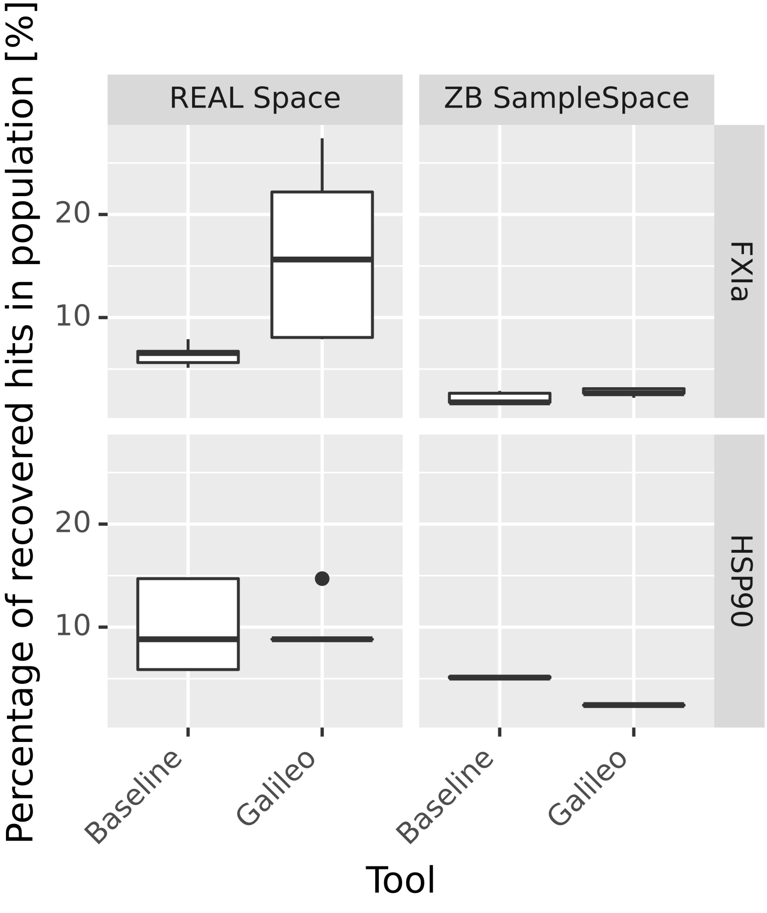


**Fig. A7** Average hit recovery for the pharmacophore search experiment. For the ZB SampleSpace, the number of unique hits for each run of Galileo and the random sampling (baseline) was compared to the number of hits in the enumerated version of the space. For the REAL Space, the corresponding numbers were compared to the total number of unique hits that were found over the course of all runs combined

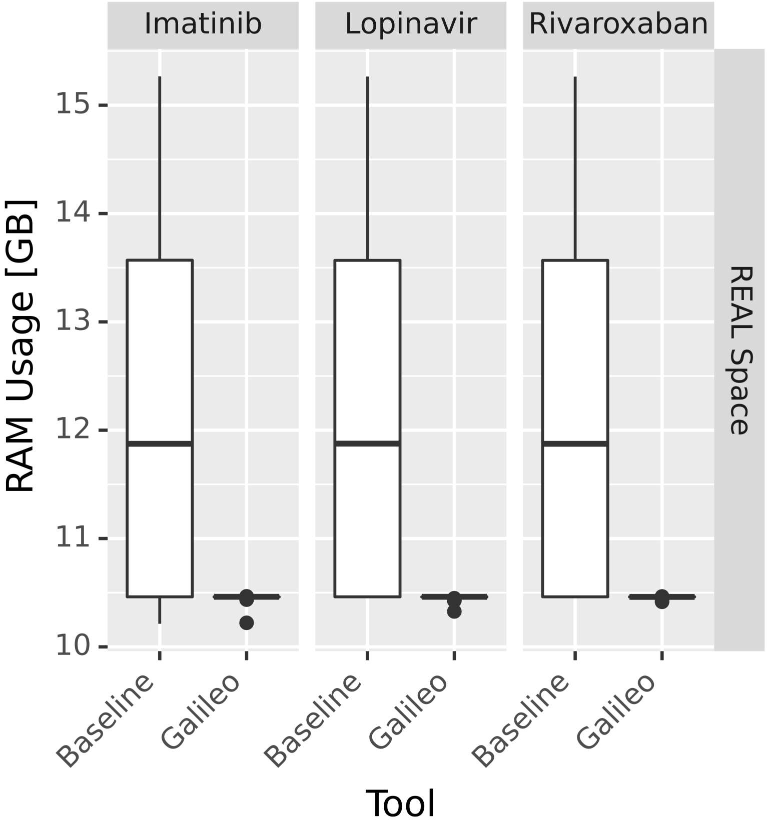


**Fig. A8** The amount of RAM that was used during the Galileo validation experiment. The RAM usage of both the regular Galileo runs and the random sampling (baseline) runs are shown for each target molecule. The values are given in GB

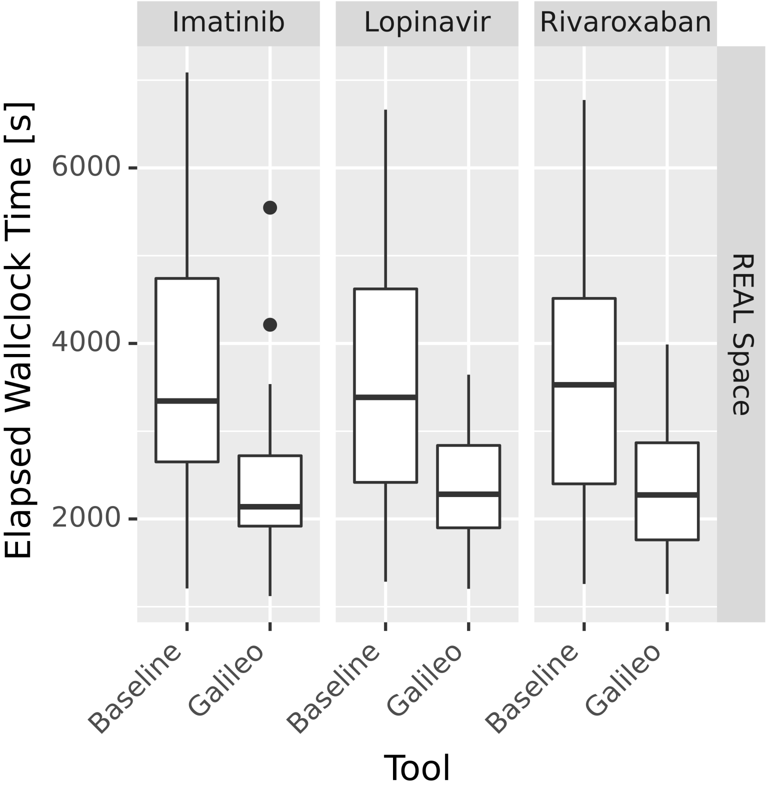


**Fig. A9** The elapsed wallclock time for the Galileo validation experiment. The elapsed time of both the regular Galileo runs and the random sampling (baseline) runs are shown for each target molecule. The values are given in seconds

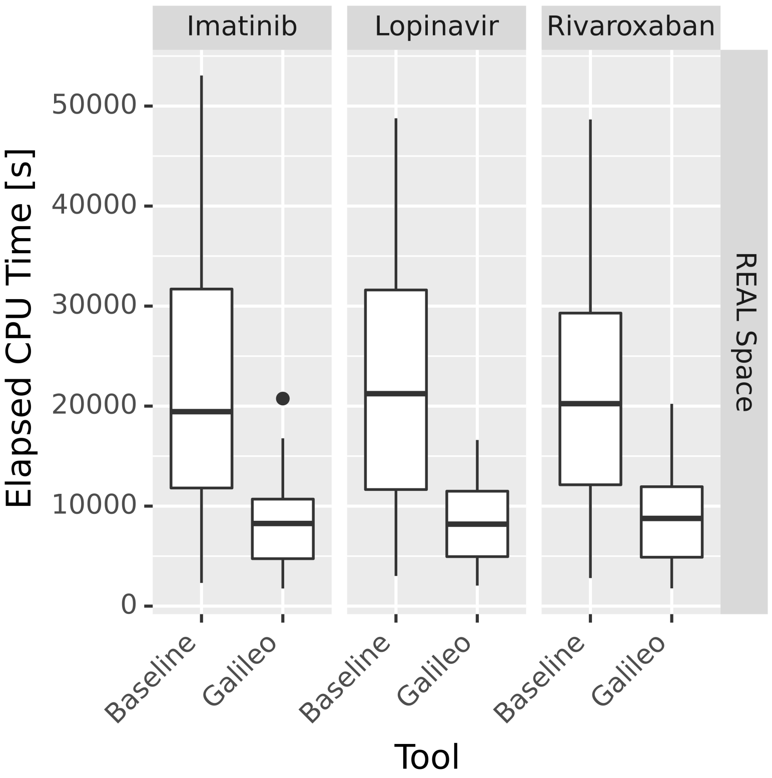


**Fig. A10** The elapsed CPU time for the Galileo validation experiment. The elapsed time of both the regular Galileo runs and the random sampling (baseline) runs are shown for each target molecule. The values are given in seconds

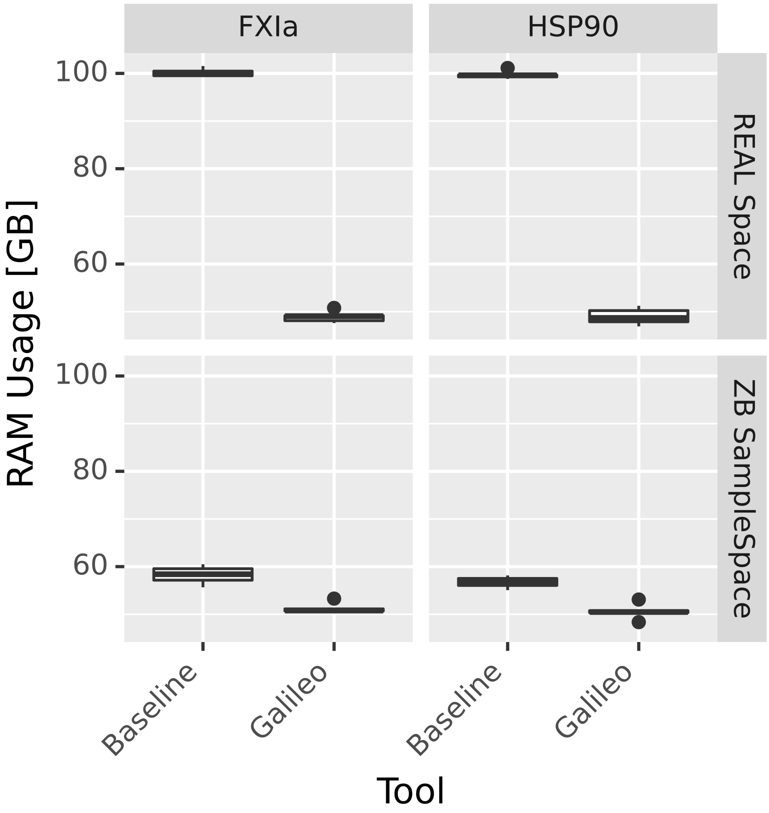


**Fig. A11** The amount of RAM that was used during the pharmacophore search experiment. The RAM usage of both the regular Galileo runs and the random sampling (baseline) runs are shown for each target pharmacophore and fragment space. The values are given in GB

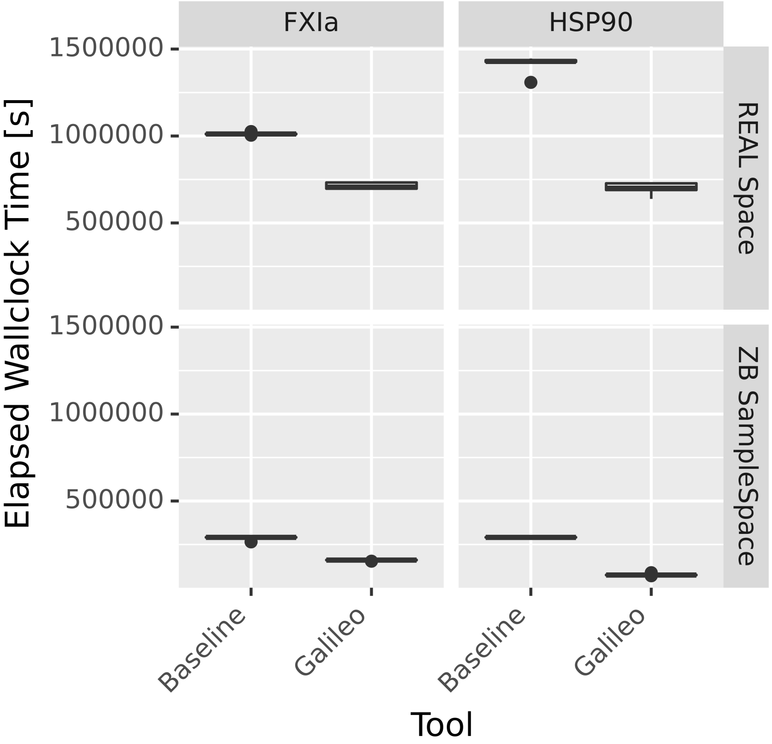


**Fig. A12** The elapsed wallclock time for the pharmacophore search experiment. The elapsed time of both the regular Galileo runs and the random sampling (baseline) runs are shown for each target pharmacophore and fragment space. The values are given in seconds

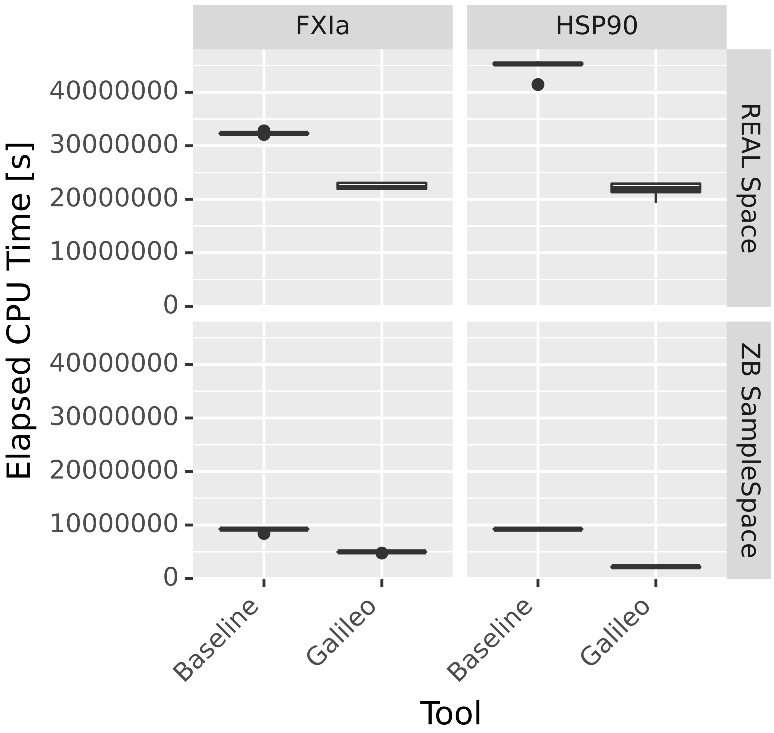


**Fig. A13** The elapsed CPU time for the pharmacophore search experiment. The elapsed time of both the regular Galileo runs and the random sampling (baseline) runs are shown for each target pharmacophore and fragment space. The values are given in seconds
